# Using wearable technology for Posture Regulation to Improve Surgical Ergonomics in the paediatric operating room: the UPRISE trial: a pilot study

**DOI:** 10.1007/s00464-024-10933-5

**Published:** 2024-06-20

**Authors:** Suvarna Soni, Angus M. A. Ritchie, Sue Liu, Maurizio Pacilli, Ramesh M. Nataraja

**Affiliations:** 1 Departments of Paediatrics and Surgery, School of Clinical Sciences, Faculty of Medicine, Nursing and Health Sciences, Monash University, Melbourne, Australia; 2 Departments of Paediatric Surgery and Monash Children’s Simulation, Monash Children’s Hospital, 246 Clayton Road, Clayton, Melbourne, 3168 Australia

**Keywords:** Ergonomics, Paediatric surgery, Posture, Wearable technology, Musculoskeletal diseases

## Abstract

**Background:**

The rising prevalence of work-related musculoskeletal disorders has numerous physical, financial, and mental repercussions for surgeons. This study aims to establish whether the use of a wearable posture device can improve the operating time spent in suboptimal, high-risk postures.

**Methods:**

Surgeons were recruited in Phase 1 of this prospective randomised study and baseline postural data was obtained. In Phase 2, participants were randomised to receive either a traditional educational workshop or intraoperative vibrations from the device to correct postural lapses. During minor elective day cases, intraoperative postural data was collected and stratified by forward flexion angle, into five risk categories (negligible to very high). Participants’ experience with the sensor was also assessed.

**Results:**

A total of 100 surgical procedures (Phase 1: n = 50; Phase 2: n = 50) were performed by eight surgeons of varying seniority. Exposure to the educational intervention increased time spent in suboptimal posture (Phase 1 vs. Phase 2); 47.5% vs. 67.8%, p = 0.05. However, the vibrational intervention significantly reduced this time; 50.0% vs. 20.7%, p = 0.005. Procedure type didn’t influence posture although, laparoscopic interventions spent most time in negligible-risk postures; 47.7% vs. 49.3%, compared to open procedures. Surgical consultants spent less time in suboptimal posture compared to fellow/registrars; 30.3% vs. 72.6% (Phase 1) and 33.8% vs. 65.3% (Phase 2).

**Conclusion:**

Vibrational intervention from the device significantly decreased the time spent in suboptimal, high-risk postures. As procedure type wasn’t correlated with postural changes, surgeon-specific factors in regulating posture are paramount. Finally, surgeon experience was positively correlated with improved surgical ergonomics.

**Supplementary Information:**

The online version contains supplementary material available at 10.1007/s00464-024-10933-5.

Work-related musculoskeletal disorders (WMSDs) are debilitating yet preventable conditions prominent in occupations requiring prolonged periods of forceful, repetitive, and awkward postures, which disproportionately affect surgeons [1]. The significant impact of this impending epidemic is further highlighted by the statistically significant association between fewer working years and developing WMSDs [2, 3]. The lack of intervention in this group is becoming increasingly apparent when juxtaposed with the predicted shortage in the surgical workforce by 2025, and the great advances in industrial ergonomics to alleviate the burden of disease [4–6]. This issue is worsened by factors such as toxic productivity, under-reporting, inadequate ergonomic education, and the one-size-fits-all design of surgical equipment [5, 7–9].

The burden of WMSDs amongst surgeons is multifaceted, as it impacts their physical, financial, emotional, and mental health. A 2019 cross-sectional study, implicated the rising prevalence of WMSDs with workplace absenteeism and loss in productivity, as 31% of surgeons reporting discomfort couldn’t work and altered their caseload [3, 10]. Moreover, the annual average loss of 7.3 days due to WMSDs, translates to approximately $41,000 in hospital revenue [11]. Alongside, the financial consequences faced by hospitals, surgeons also lose income and risk early retirement [12, 13]. Furthermore, the persistence of musculoskeletal (MSK) pain outside the operating room (OR), directly deteriorates surgeons’ mental and emotional wellbeing. In two multi-centre cohort studies, 51% of participants attributed interruptions in their relationships and sleep deprivation to their pain [13, 14]. Subsequently, the sleep deprivation induced cognitive decline can increase surgical error risk and compromises patient safety [14]. The cumulative effects of these disruptions with a poor work-life balance has been implicated in surgeon burnout [13, 15].

Current published literature focuses on potential perioperative ergonomic interventions which modify extrinsic factors within the OR. This includes, monitor placement [16, 17], operating table height [18, 19], foot pedal placement [20, 21], instrument handles [22–24], and body-support chairs/surgical platforms [25, 26]. However, the heterogeneity in the efficacy of equipment-based interventions corroborate the conclusion that it is extremely challenging to account and address every dynamic factor within the OR. In addition, there is a paucity of surgeon-focused interventions to improve ergonomics within the surgical operating theatre.

In addition to the rise in prevalence of WMSDs, the ergonomic challenges within the OR are exacerbated by inadequate awareness and education regarding proper OR setup and surgeon posture [27]. Thus, it is vital to alter the trajectory of current research towards surgeon-focused interventions, the only constant within the dynamic OR environment. Furthermore, by identifying and validating tools that rely on internal drivers rather than external factors, effective long-term change is more likely.

Coincidentally, wearable technology is becoming more widespread in many aspects of daily life and there is increasing usage in the operating room [28]. Considering the effectiveness of internal drivers of change, the absence of interventions to rectify poor posture and the advent of wearable technology, a promising area of research can be identified. This is addressed with our study which aims to investigate how intraoperative postural regulation using wearable technology can help improve ergonomics within the paediatric operating theatre.

## Methodology

### Study design

Prospective randomised study conducted at a tertiary paediatric surgical institution. There were two phases to the study. In Phase 1, participants’ baseline posture was captured. In Phase 2, participants were randomised using an online randomizing software, into either the educational or vibrational intervention group [29]. Surgeons in the education group were observed after receiving an educational workshop about the importance of posture with suggestions to improve intraoperative posture. In the vibration group, surgeons received intraoperative vibrations from the wearable UPRIGHT GO 2 (Upright Technologies, Israel) posture sensor to correct postural lapses. Posture lapses exceeding the threshold, triggered the vibrations after a 5-s delay. The sensor continued to vibrate until the participant returned to a good posture.

### Participants

Surgeons with differing levels of expertise and competency were recruited from the department. Recruitment was voluntary and confirmed prior to each procedure. Consent was also obtained from the patient and/or parent/guardian after providing a verbal explanation with the scope of the impact of this research. There was no change to the operative intervention for the patient.

### Operative procedures

The included procedures were standardised to minor elective day cases (e.g., hernia/hydrocele repairs, orchidopexies, orchidectomies, circumcisions) and laparoscopic appendectomies. Hence, the surgeons were randomised as they were the only variable factor. The selection of paediatric surgeries was primarily driven by its practicality and accessibility as this study was conducted in a tertiary paediatric hospital. To assess the outlined aims and control for procedure duration, emergency (e.g., abscess drainage) and longer, major procedures (e.g., laparotomies) were excluded. A supplementary laparoscopic-only analysis was also conducted to determine the effect of each intervention on surgeon posture.

### Baseline survey

An electronic preliminary participant questionnaire (Qualtrics, Sydney NSW) determined the participants’ demographics including gender, height, frequency of physical activity, surgical experience & history of MSK pain/discomfort [30]. This survey was constructed as a modification of the Cornell Musculoskeletal Discomfort Questionnaire for standing workers [31].

### Educational intervention

An educational workshop session was designed focusing on the importance of good posture, consequences of poor posture, and simple adjustments to ensure optimal ergonomics are maintained. An educational package with additional resources was distributed electronically after the session.

### Intraoperative data collection

**Fig. 1:**
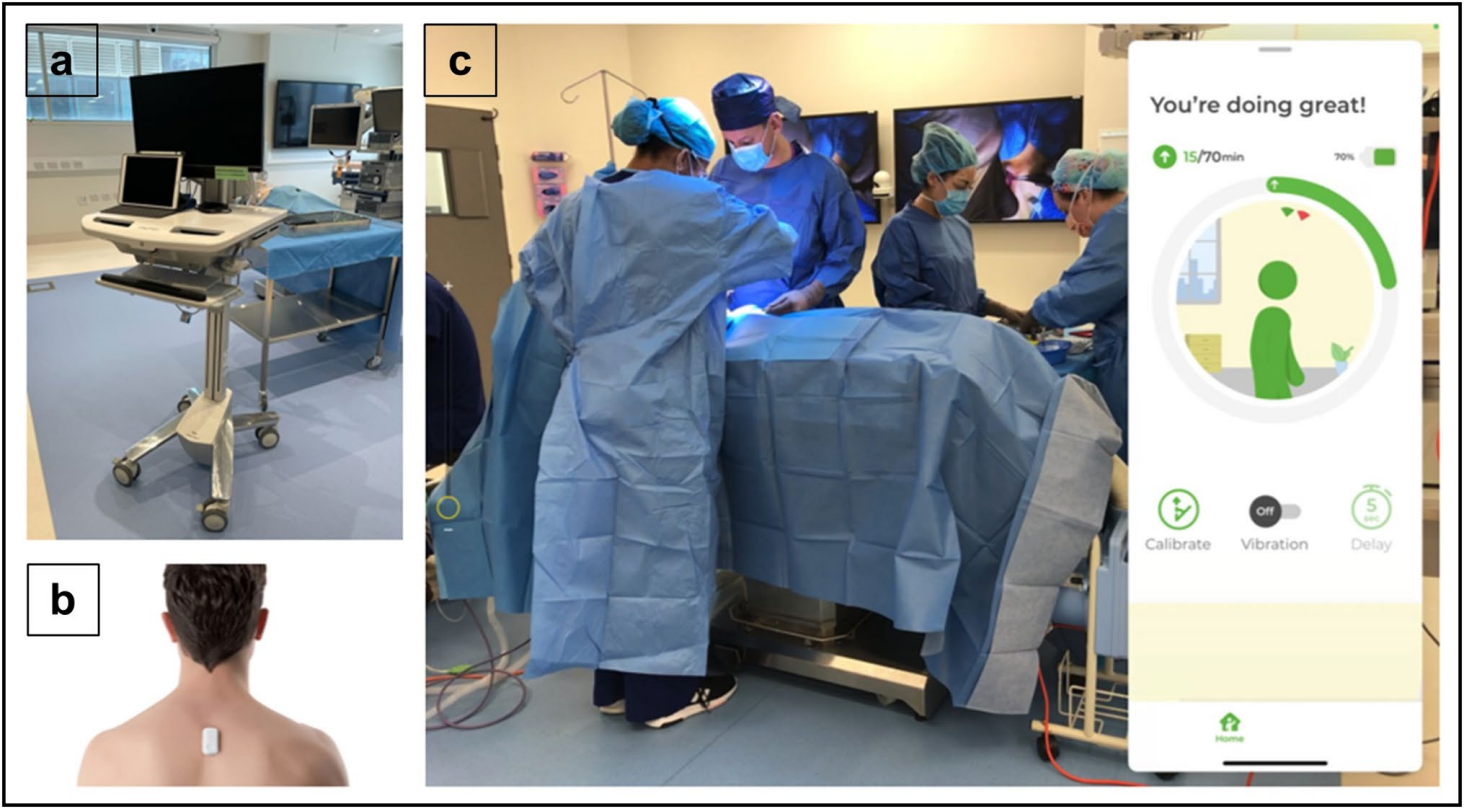
**a** Mobile Computer Workstation. **b** UPRIGHT GO 2 placement. **c** Workstation Display – theatre environment and UPRIGHT GO 2 display

A dedicated mobile computer workstation was constructed (Fig. 1a). Sensor positioning and calibration in a neutral, upright position occurred prior to the procedure (Fig. 1b). The workstation captured the theatre environment and the live UPRIGHT GO 2 (Upright Technologies, Israel) display (Fig. 1c). The surgeon’s activity and posture were recorded at 10-s intervals and then used to calculate the percentage of operating time (%OT) the surgeon spent in suboptimal posture. Time taken for swapping instruments, standing, talking and/or fixing equipment was excluded from this assessment. The procedure commenced with the first incision and finished with the final suture placement. The range of good posture was determined to be the 4th setting (~ 19.83° away from the vertical), based on the ideal surgical posture.

The ergonomic assessment tool of Rapid Entire Body Assessment was modified to evaluate the risk of developing MSK disorders associated with varying trunk postures [32]. These modifications were made within the constraints of the posture sensor’s abilities (i.e., trunk extension, lateral flexion, forward flexion > 60°). The surgeon’s posture was quantified by measurement of the deviation of the surgeon from an upright posture. To describe what the level of risk of developing MSK disorders was if that posture was maintained, the angles of deviation were stratified into 5 categories:

1. 0° to 10° = Negligible risk
2. 10° to 20° = Low risk
3. 20° to 30° = Moderate risk
4. 30° to 40° = High risk
5. 40° to 50**°** = Very high risk

As this is a pilot study, the intervention and endpoints are not validated. However, studies have shown that using real-time biofeedback allows participants to minimise time spent in end-range lumbar spine flexion, which in turn is vital in mitigating MSK issues [33–35].

Additional data collected intraoperatively included: foot pedal/loupes usage, table adjustment, procedure duration, and type of procedure.

### Postoperative discomfort questionnaire

Surgeon experience with the sensor was captured with a questionnaire utilising a 5-point Likert Scale ranging from 1 (strongly agree) to 5 (strongly disagree). Experience, performance, awareness, and stress domains were assessed.

### Power calculation

This pilot study is the first to correlate postural data with type of surgery and level of surgical experience. Due to the lack of other comparison trials, a power calculation was not possible. Therefore, we chose a convenient sample of 50 surgical procedures per phase which were deemed to be feasible within the timeframe of the available resources and personnel.

### Statistical analysis

Statistical analysis was conducted with GraphPad Prism 9.1.2 (GraphPad Software, La Jolla California, USA).

Data are reported as median values. Data was analysed by Shapiro–Wilk test, Mann–Whitney *U* tests, Kruskal–Wallis tests, Friedman tests, and one-way ANOVA with post-hoc Tukey’s multiple comparisons tests as appropriate.

A two-sided p-value of 0.05 was considered statistically significant.

### Human research ethics committee approval

Human Research Ethics Committee (HREC) approval was obtained from Monash University (2021-29022-63575) and Monash Health (RES-21-0000-158L).

## Results

### Participant demographics

A total of 8/11 (72.7%) eligible surgeons were formally recruited to the study which was conducted from May 2021 and ended in July 2022. Of these, 62.5% were female, and the mean height of the participants was 166 ± 10.85 cm. There were no significant differences between the participants receiving the educational (n = 4) and vibrational (n = 4) intervention, Table S1. No data was missing for any outcome for any participant.

### Surgical procedures

A total of 107 surgical procedures were performed by the eight recruited surgeons. Seven of these surgical procedures were excluded in Phase 1; sensor recalibration issues (n = 6), and technical failures for screen-recording (n = 1). Therefore, as summarised by the CONSORT diagram in Fig. S1, a total of 100 procedures were included in the final analysis with 50 procedures in each phase.

### Educational vs. vibrational interventions

**Fig. 2:**
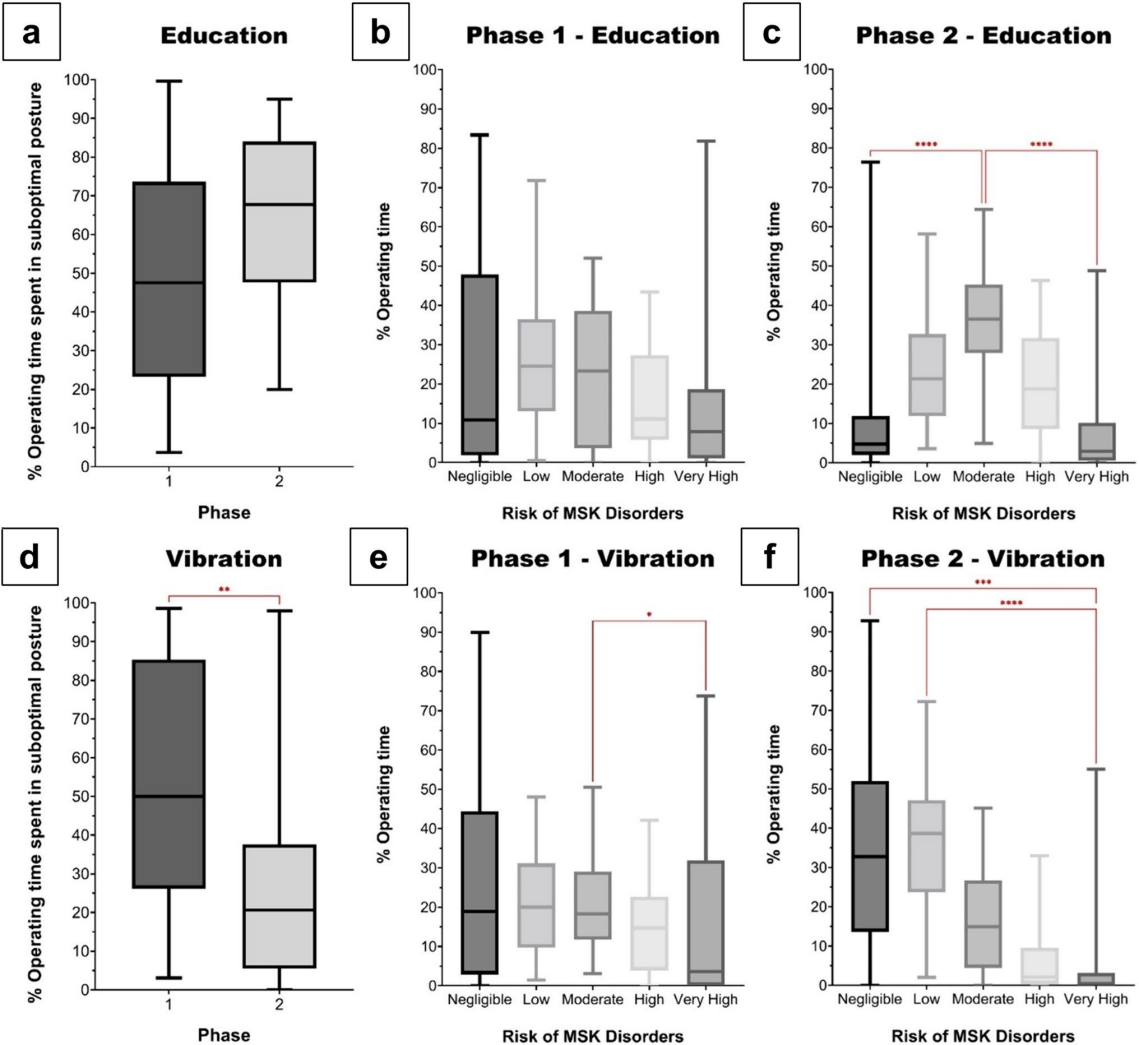
Time spent in suboptimal posture in participants receiving an **a** educational intervention **d** vibrational intervention. Distribution of musculoskeletal disorder risk amongst participants in the educational **b** Phase 1, **c** Phase 2; and vibrational **e** Phase 1, **f** Phase 2 intervention (*p = 0.04, **p = 0.005, ***p = 0.001, ****p < *0.0001*)

After exposure to an educational intervention, participants spent more %OT in suboptimal postures (47.5% vs. 67.8%, p = 0.05), Fig. 2a. Similarly, although participants spent majority of %OT in low-risk postures in Phase 1 (n = 19) (Fig. 2b), this deteriorated to moderate-risk postures in Phase 2 (n = 32) (Fig. 2c). Conversely, with vibrational intervention, the %OT spent in suboptimal postures reduced significantly (50.0% vs. 20.7%; p = 0.005), Fig. 2d. Prior to any intervention, participants (n = 31) spent more time in moderate-risk postures, as compared to very high-risk postures (Fig. 2e). However, after exposure to the vibrational intervention participants (n = 18) spent significantly more %OT in negligible/low-risk postures, as compared to very-high risk postures (Fig. 2f).

### Laparoscopic vs. open procedures

**Fig. 3:**
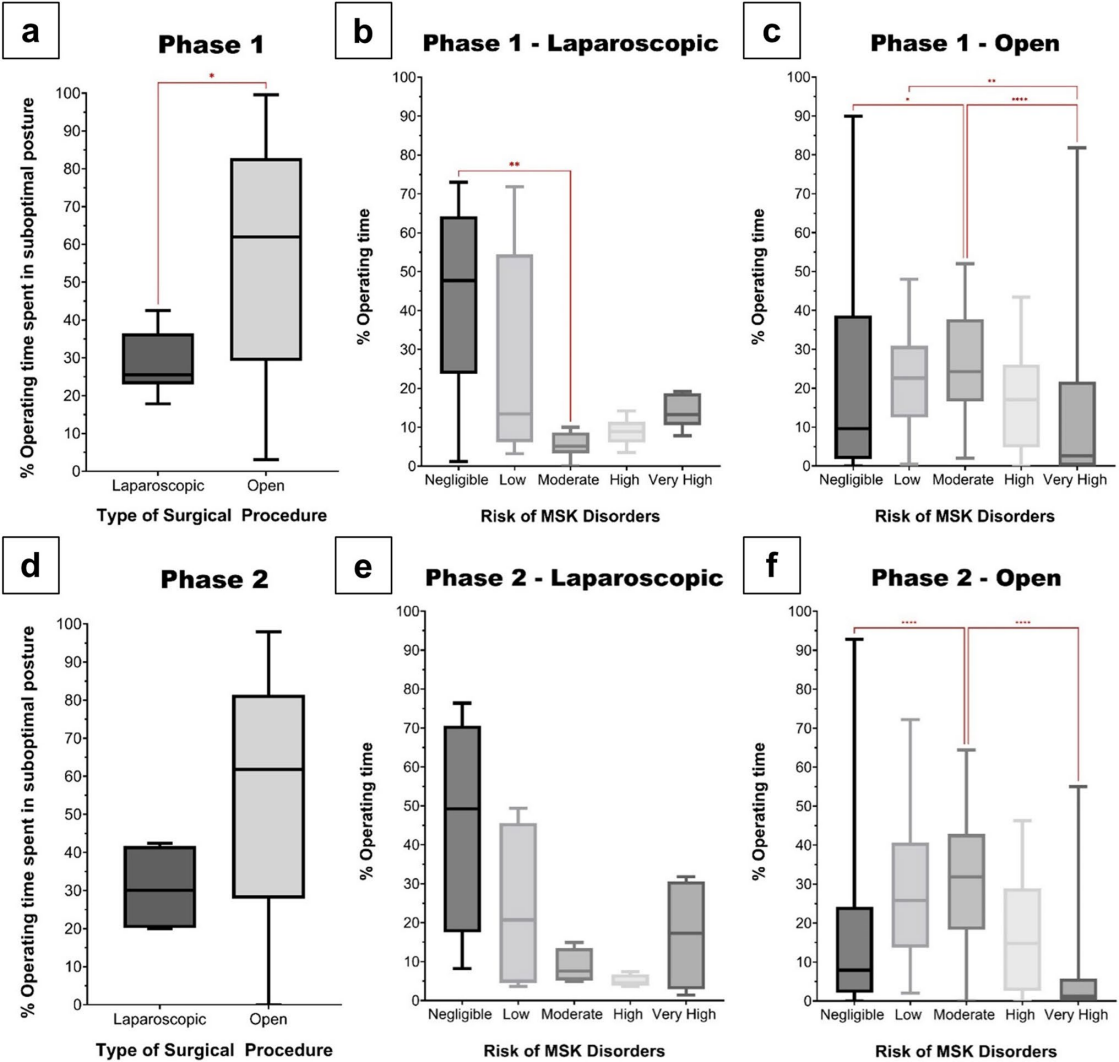
Time spent in suboptimal posture amongst participants performing laparoscopic and open procedures for **a** Phase 1 and **d** Phase 2. Distribution of musculoskeletal disorder risk amongst participants performing laparoscopic procedures in **b** Phase 1, **c** Phase 2; and open procedures in **e** Phase 1, **f** Phase 2 (*p = 0.01, **p = 0.002, ****p < *0.0001*)

In Phase 1, the %OT spent in suboptimal posture was significantly (p = 0.02) less in laparoscopic compared to open procedures; 25.5% (n = 8) vs. 62.0% (n = 42), Fig. 3a. Furthermore, participants operating laparoscopically spent significantly (p = 0.002) more time in negligible vs. moderate risk postures; 47.7% vs. 5.1% (Fig. 3b). Conversely, participants performing open procedures were mostly at moderate risk (24.3%) compared to negligible (9.6%) or very high (2.6%) risk postures (Fig. 3c).

In Phase 2, the %OT spent in suboptimal posture was lower in laparoscopic compared to open procedures; 30.1% (n = 4) vs. 61.8% (n = 46), Fig. 3d, this did not reach significance (p = 0.1). Exposure to interventions didn’t alter the risk distribution, as laparoscopic procedures (Fig. 3e) were mostly performed in negligible risk postures (49.3%), and open procedures (Fig. 3f) in moderate risk postures (31.9%).

### Supplementary laparoscopic-only analysis

After exposure to an educational intervention, participants spent more %OT in suboptimal postures (23.6% vs. 39.5%, p = 0.4), Fig. S2a. Nevertheless, participants spent majority of %OT in negligible-risk postures in both Phase 1 (n = 6) (Fig. S2b) and Phase 2 (n = 3) (Fig. S2c). Conversely, exposure to the vibrational intervention, reduced the %OT spent in suboptimal postures (36.7% vs. 20.6%), Fig. S2d. However, the significance of this change cannot be validated due to the small sample size. Similar to the educational intervention, all participants spent majority of %OT in negligible risk postures in both Phase 1 (n = 2) (Fig. S2e) and Phase 2 (n = 1) (Fig. S2f).

### Effect of surgeon experience

**Fig. 4:**
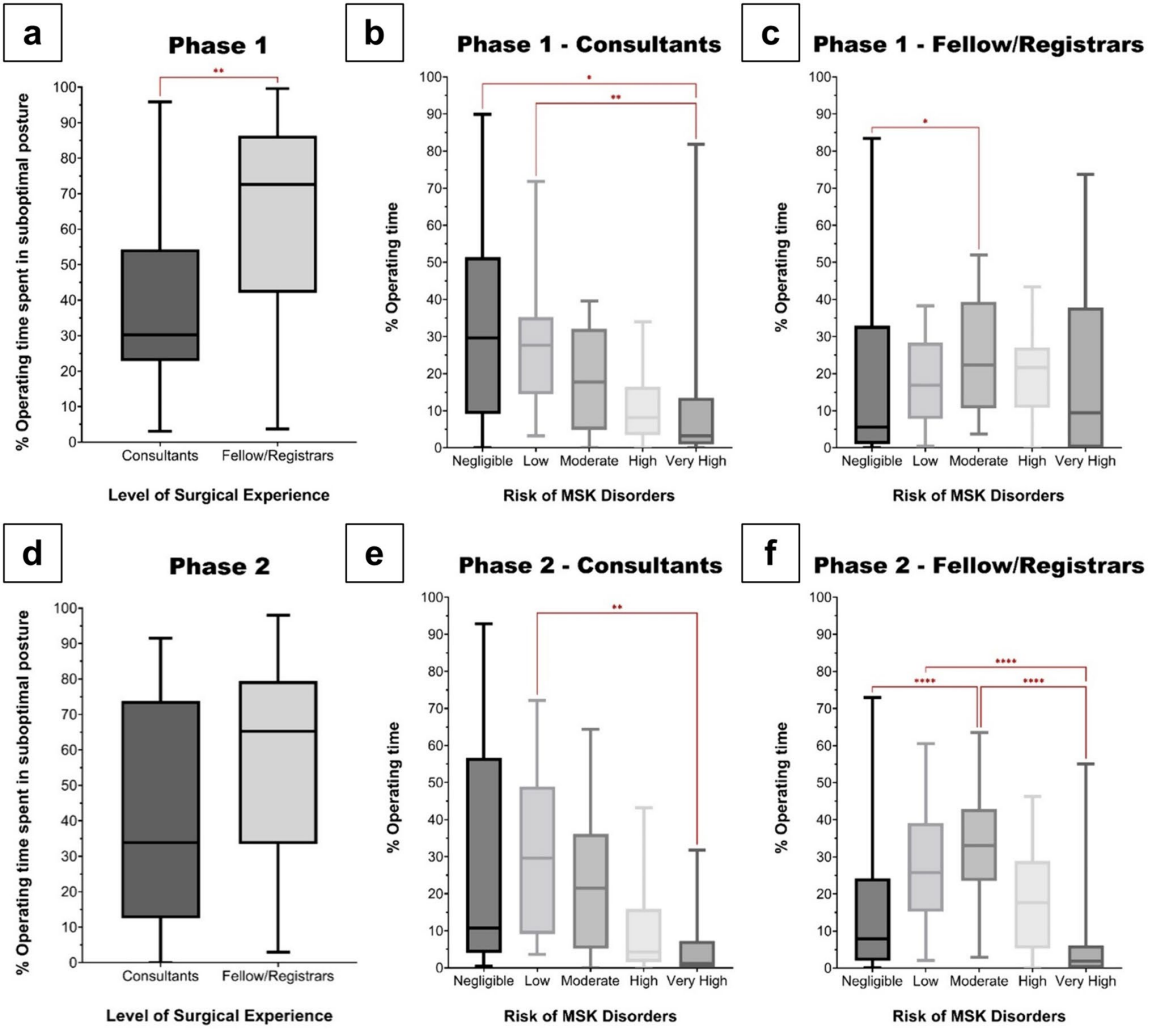
Time spent in suboptimal posture by consultants and fellow/registrars in **a** Phase 1, and **d** Phase 2. Distribution of musculoskeletal disorder risk between consultants **b** Phase 1, **e** Phase 2; and fellow/registrars **c** Phase 1, **f** Phase 2 (**a** **p = *0.004*. **b** *p = 0.01, **p = *0.001.* **c** *p = *0.03*. **e** **p = *0.005*. **f** ****p < *0.0001)*

In Phase 1, the %OT spent in suboptimal posture was significantly lower in consultants compared to fellow/registrars; 30.3% (n = 24) vs. 72.6% (n = 26), p = 0.004, Fig. 4a. Consultants spent significantly more time in negligible (29.6%; p = 0.01) or low-risk (27.7%; p = 0.001) postures vs. very high-risk (3.2%) postures (Fig. 4b). Alternatively, fellow/registrars spent significantly (p = 0.03) more time in moderate (22.4%) vs. negligible-risk (5.6%) postures (Fig. 4c).

In Phase 2, whilst consultants spent less %OT in suboptimal posture compared to fellow/registrars, this was not significant; 33.8% (n = 16) vs. 65.3%; (n = 34), p = 0.054 (Fig. 4d). Consultants spent significantly (p = 0.005) more time in low (29.6%) vs. very high-risk (1.2%) postures (Fig. 4e). Conversely, fellow/registrars spent significantly (p < 0.0001) more time in low (25.8%) or moderate-risk (33%) postures, as compared to negligible (7.9%) or very high-risk (1.9%) postures (Fig. 4f). In both phases, there were no significant differences in the overall risk distributions between consultants and fellow/registrars.

### Different types of surgical procedures

Between Phase 1 and 2, there were no significant difference between the proportion of laparoscopic (16% vs. 8%, p = 0.5) and open procedures performed (84% vs. 92%, p = 0.9), Table S2.

**Fig. 5:**
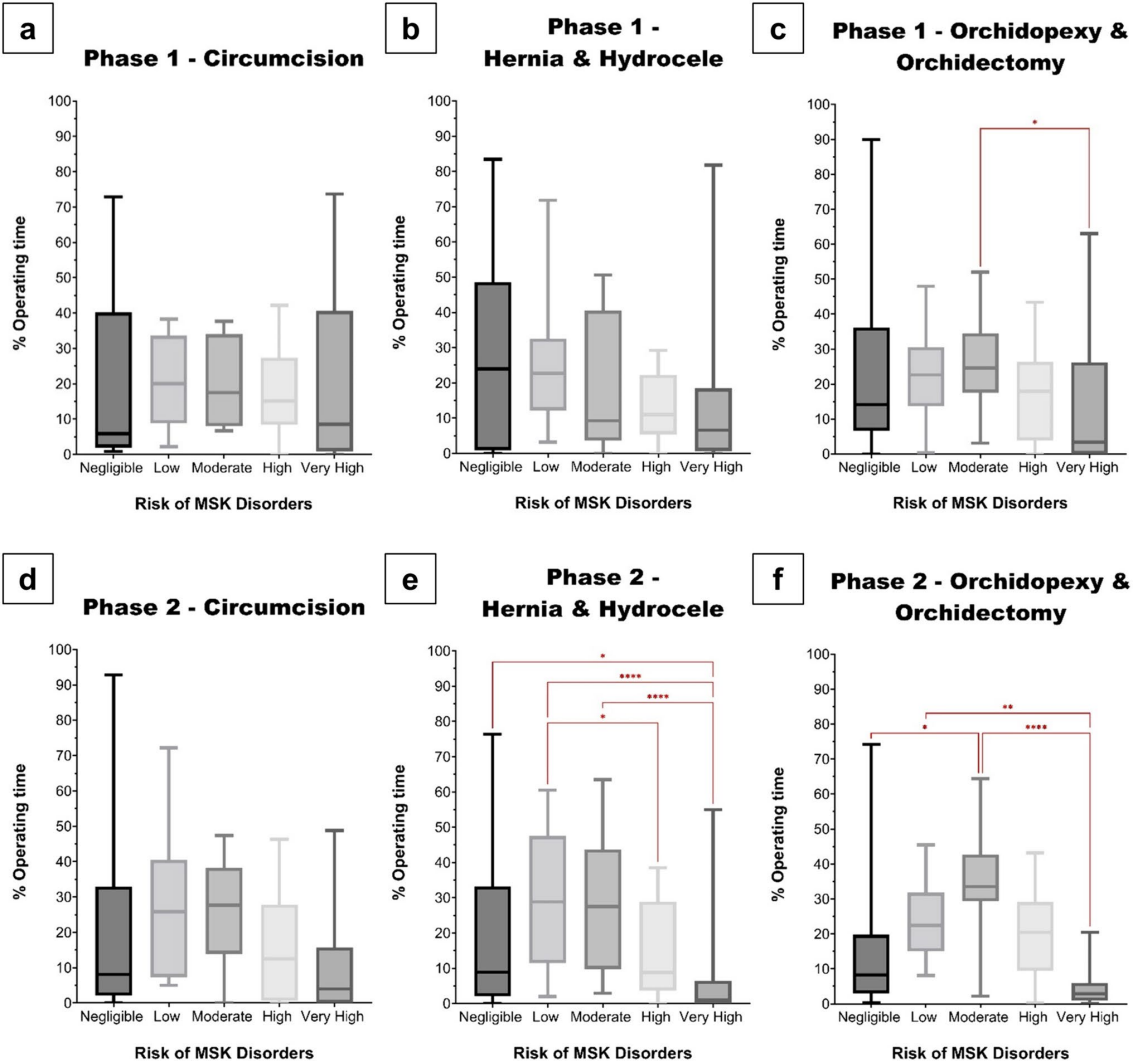
Distributions of musculoskeletal disorder risk when performing circumcisions (**a** Phase 1 and **d** Phase 2), hernias/hydroceles (**b** Phase 1 and **e** Phase 2), and orchidopexies/orchidectomies (**c** Phase 1 and **f** Phase 2). **c** *p = *0.04*. **e** *p = 0.03, ****p < *0.0001*. **f** *p = 0.02, **p = 0.004, ****p < *0.0001*

The type of surgical procedure did not significantly affect the %OT spent in suboptimal posture both within and between each phase. In both Phase 1 and 2, there were no significant differences between the mean %OT spent in suboptimal posture when performing a circumcision (60.3% vs. 51.5%, p = 0.5), hernia/hydrocele (45.8% vs. 49.4%, p = 0.7), or orchidopexy (55.1% vs. 59.0%, p = 0.7). Participants operated mostly in low (Phase 1) or moderate-risk (Phase 2) postures when performing circumcisions (Fig. 5a and d), and in negligible (Phase 1) or low-risk (Phase 2) postures whilst performing a hernia/hydrocele repair (Fig. 5b and e). However, when performing an orchidopexy, moderate-risk postures were maintained for majority of the operating time (Fig. 5c and f). There were no significant differences in risk distribution between the procedures, across both phases.

### Assessment of sensor experience

For both interventional groups, there were no significant differences between majority of the outlined domains, assessing the experiences of the sensors, Table 1. The only exception was seen in Phase 1, where participants receiving the educational intervention agreed the sensor was comfortable. However, in Phase 2, this changed to strongly agreed (*p* = 0.002).

**Table 1:** Effect of educational vs. vibrational intervention on participant experience, rated on a Likert scale from 1 (strongly agree) to 5 (strongly disagree)

Domain		Educational Intervention (n = 4)			Vibrational Intervention (n = 4)		
		Phase 1	Phase 2	p-value	Phase 1	Phase 2	p-value
Experience & performance							
1	I had a positive experience with the sensor	2 (1–2)	2 (1–3)	0.5	2 (1–3)	2 (1–4)	0.3
2	The sensor was comfortable	2 (1–2)	1 (1–2)	**0.002	2 (1–4)	2 (1–4)	0.5
3	The sensor was painful	5 (2–5)	5 (4–5)	0.07	4 (1–5)	4 (3–5)	0.06
4	The sensor influenced my performance positively	3 (2–3)	3 (2–4)	0.17	3 (2–5)	3 (1–4)	0.2
Awareness							
1	I was more aware of my posture during surgery	2 (1–4)	2 (2–4)	0.7	2 (1–4)	2 (2–4)	0.4
2	I was more distracted by my posture during surgery	4 (2–5)	4 (2–5)	0.7	4 (2–4)	4 (2–4)	0.9
Stress							
1	I was stressed whilst performing the surgery	4 (2–5)	4 (1–5)	0.05	4 (2–5)	4 (2–5)	0.9
2	There were unexpected events or complications during the surgery	4 (2–5)	5 (1–5)	0.3	4 (2–5)	4 (2–5)	0.7

Discrete data is presented as median (range)

## Discussion

In this prospective randomised interventional study for surgeon posture although baseline %OT spent in suboptimal posture was similar in all participants, posture improved significantly after the continuous vibrational intervention and deteriorated after the educational intervention. Participants receiving the educational intervention spent more time in moderate risk postures than very high-risk postures, indicating some postural improvement. Although the deterioration of posture in participants receiving the educational intervention was unexpected, it may be explained by the lack of individualisation of a passive method. This can subsequently reduce engagement with and reinforcement of ergonomic principles, ultimately leading to insufficient drivers for long-term change in posture habits [36, 37]. Conversely, the approximately even risk distribution of participants receiving the vibrational intervention, became positively skewed in Phase 2, where they operated mostly in negligible/low-risk postures. The sensor was tolerated by all the participants who predominantly agreed that the sensor was comfortable and that they had a positive experience with it. The sensor also had no impact on their performance. Given the novel nature of this area of research, there are no other studies exploring the effectiveness of a vibrational intervention in improving surgical ergonomics. However, a recent systematic review analysing the effect of ergonomics training outside the OR and intra-operative microbreaks revealed that 70% of surgeons felt an improvement in their symptoms after undergoing ergonomics training [38]. This study provides a promising avenue of future research as we have demonstrated that vibrational interventions for postural regulation are more effective than education alone. Larger-scale studies across different surgical sub-specialties will further validate its efficacy. This short-term intervention could provide substantial long-term benefits in improving prevalence of WMSDs amongst surgeons.

Participants spent less %OT in suboptimal posture when performing laparoscopic procedures as compared to open procedures in both phases, although the small sample size limited this analysis. Laparoscopic procedures allowed participants to operate mostly in negligible risk postures, whereas open procedures required moderate-risk postures. Therefore, laparoscopic procedures may facilitate improved surgical ergonomics as open procedures necessitate greater neck and torso flexion [39–41], whilst laparoscopic procedures provide greater ergonomic benefit and place less load on the neck [42, 43]. However, there is evidence that minimally invasive surgeons are more at risk of experiencing MSK symptoms, as laparoscopy induced greater forearm muscle activation and discomfort as compared to open procedures [44–47]. This may not reflect the true ergonomic benefit of laparoscopy, as the opposing evidence is predominantly subjective, outdated, and potentially influenced by incorrect posture with the initial introduction of laparoscopy. Additionally, the STORZ OR1 theatres (KARL STORZ, Australia) in our institution, may have influenced our results as they allow equipment to be adjusted based on the ideal ergonomic setup, as opposed to the historical variants [48].

To further validate these results, a retrospective supplementary analysis was conducted on the laparoscopic group. However, it is important to highlight that since the sample size was very small (Phase 1: n = 8; Phase 2: n = 4), statistical analysis was not possible on some results and other analyses may be underpowered. Nevertheless, future studies will be designed to ensure data from open and laparoscopic procedures are collected equally, to ensure a more meaningful comparison. Consultants were hypothesised to have potentially worse posture than fellow/registrars secondary to ingrained habits/practices and increased age demographic which could infer an increased susceptibility to MSK problems, irrespective of the workplace [49]. However, in both phases, consultants spent less time in suboptimal postures than their junior counterparts. Moreover, whilst consultants mostly operated in negligible/low-risk postures, fellow/registrars spent majority of the %OT in moderate-risk postures. This is likely due to increased experience and procedural competency leading to less cognitive load, better subconscious maintenance of posture, and increased conscious focus diversion onto factors beyond technical skills. Additionally, fellow/registrars may focus primarily on the procedure itself, which detracts from the attention they direct towards maintaining posture. Numerous studies focusing on laparoscopy, endoscopy, and robotic surgery, substantiate the positive influence of previous surgical experience on the surgeons’ adopted postures [50–52]. Though they can be extrapolated to open procedures, further research is needed to confirm this. Postural programs for junior surgeons are necessary to maximise the clinical benefit of these findings. In addition, supervising surgeons should incorporate ergonomic education whilst instructing the future generation of surgeons.

Regardless of the type of procedure performed, there were no significant differences in the time spent in suboptimal postures and the risk distribution, between and within phases. Thus, poor posture can be attributed to surgeon-specific factors rather than procedure-specific ones based on our study. To limit bias and maintain continuity in our study, we restricted the elective procedures to primarily inguinal and penile procedures. However, most of the current literature focuses on comparing broad categories of surgical procedures (i.e., open, laparoscopic, endoscopic, robotic). Additionally, the degree of time spent in suboptimal posture may be directly correlated with the location of operative field and the effects of patient positioning [17, 53, 54]. Although postural awareness may be sacrificed during more stressful or complex procedures, surgeons should aim to increase mindfulness of posture irrespective of the type of procedure they are performing.

The prospective nature of our study allowed incorporation of both an observational and randomised interventional aspect. Whilst considerable research identified the rising prevalence of WMSDs amongst surgeons, there is minimal research around clinical interventions improving surgical ergonomics. Furthermore, given that the unit cost of these sensors ranges from $40-$80 AUD, this sensor is an affordable and viable intraoperative intervention which can improve surgical ergonomics. Due to the nature of the intervention, the absence of blinding in participants, assessors and study coordinators is a significant potential limitation. Other limitations arose from the sensor itself, which needed to be secured to the participants’ backs via adhesives and then calibrated to establish the normal vertical axis. This process may have been subjective and non-uniform as it was influenced by the researcher attaching the sensor and the participant’s posture at the time of calibration. However, standardisation of the process by using anatomical landmarks to guide the sensor placement, limited any potential impacts. Although, issues with Bluetooth connectivity during recording time did occur, wired devices were not feasible within the sterile OR environment. Finally, the sensor’s inability to account for lateral, backward, or forward (> 60°) bending, and the compensatory deterioration in neck posture, couldn’t be addressed as this was outside the scope of the posture sensor’s manufactured purpose. Additionally, by using only one sensor, this study primarily focuses on the impact of forward flexion on the lumbar spine. As a result, this may compromise the cervical spine by provoking suboptimal neck flexion. Hence, to mitigate the risk of MSK disorders in the neck, use of adjuncts such as loupes or a laparoscopic approach may be used as appropriate. Despite this limitation, the use of only one sensor minimises its impact on the surgeon or the procedure, and is more cost-effective.

Future investigations should consider exploring the effects of frequently used adjuncts (i.e., operating loupes, foot pedals) on posture, which can guide future ergonomic training guidelines/programs. Moreover, larger cohort studies focusing on more diverse and longer operative procedures and surgical subspecialties may help develop the foundation of this intervention being implemented into routine surgical practice. This will also allow assessment of the correlations between operative field location and postural outcomes. Lastly, investigating the impact of patient weight or incision size of the operative field on surgeon posture may also broaden the current knowledge.

## Conclusion

WMSDs are a rapidly increasing cause of concern amongst the global surgical community. In our study, vibrational interventions with wearable sensors were more effective in reducing the time participants spend in suboptimal postures. Although the type of procedure didn’t influence posture, laparoscopic procedures allowed significantly better postures than open procedures. Surgical experience was also positively correlated with postural awareness.

### Supplementary Information

Below is the link to the electronic supplementary material.

Supplementary file1 (JPG 139 KB) Table S2: Distribution of surgical procedures performed. Discrete data is presented as n (%). p-values were calculated using Mann-Whitney *U* tests for non-normally distributed data (green). p-values were calculated using unpaired t-tests for normally distributed data (blue).

Supplementary file2 (JPG 60 KB) Figure S1: CONSORT flow diagram for the UPRISE Trial

Supplementary file3 (DOCX 17 KB) Table S1: Participant Demographics. Discrete data is presented as n (%). p-values were calculated using Mann-Whitney *U* tests for non-normally distributed data (green). p-values were calculated using unpaired t-tests for normally distributed data (blue)

Supplementary file4 (JPG 317 KB) Figure S2: Laparoscopic-only Subset Analysis. Time spent in suboptimal posture in participants receiving an **a** educational intervention **d** vibrational intervention. Distribution of musculoskeletal disorder risk amongst participants in the educational **b** Phase 1, **c** Phase 2; and vibrational **e** Phase 1, **f** Phase 2 intervention for each phase (*p=0.02)
