# Supplementary figures and images for: Using wearable technology for Posture Regulation to Improve Surgical Ergonomics in the paediatric operating room: the UPRISE trial: a pilot study
Source: Surg Endosc. 2024 Jun 20;38(8):4445–56. doi: 10.1007/s00464-024-10933-5 (PMC11289160; doi:10.1007/s00464-024-10933-5)

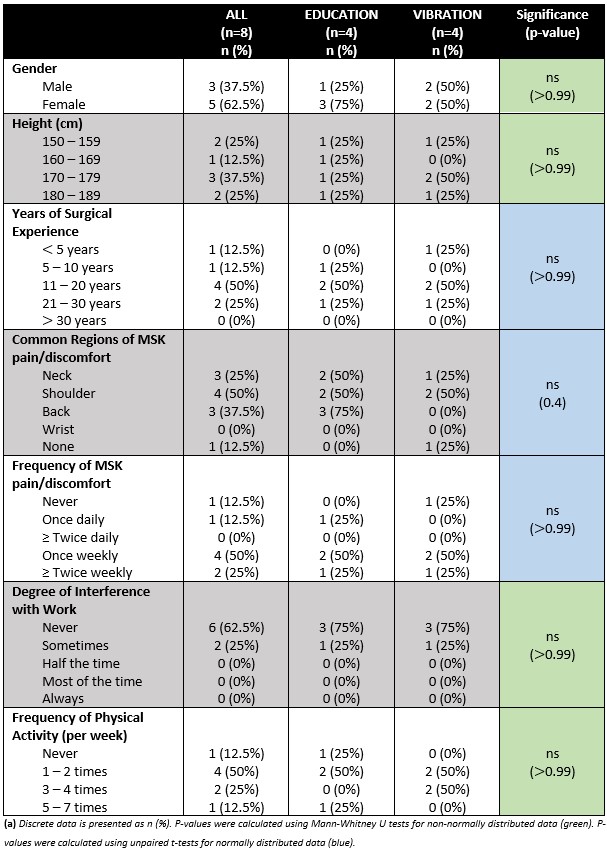

Supplement: Supplementary file 1 — Supplementary file1 (JPG 139 KB) Table S2: Distribution of surgical procedures performed. Discrete data is presented as n (%). p-values were calculated using Mann-Whitney U tests for non-normally distributed data (green). p-values were calculated using unpaired t-tests for normally distributed data (blue). [file 464_2024_10933_MOESM1_ESM.jpg]

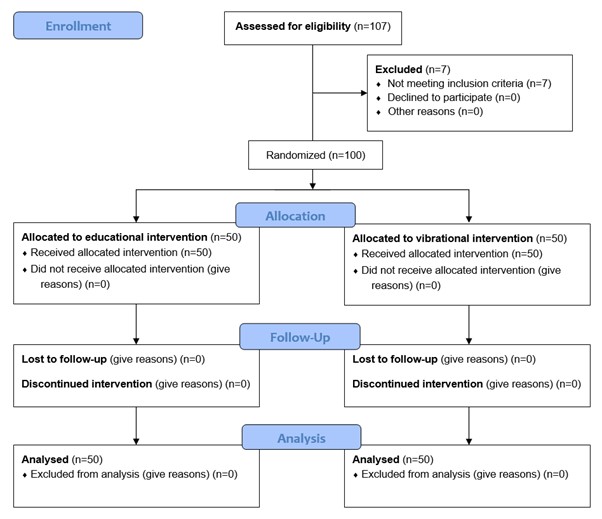

Supplement: Supplementary file 2 — Supplementary file2 (JPG 60 KB) Figure S1: CONSORT flow diagram for the UPRISE Trial [file 464_2024_10933_MOESM2_ESM.jpg]

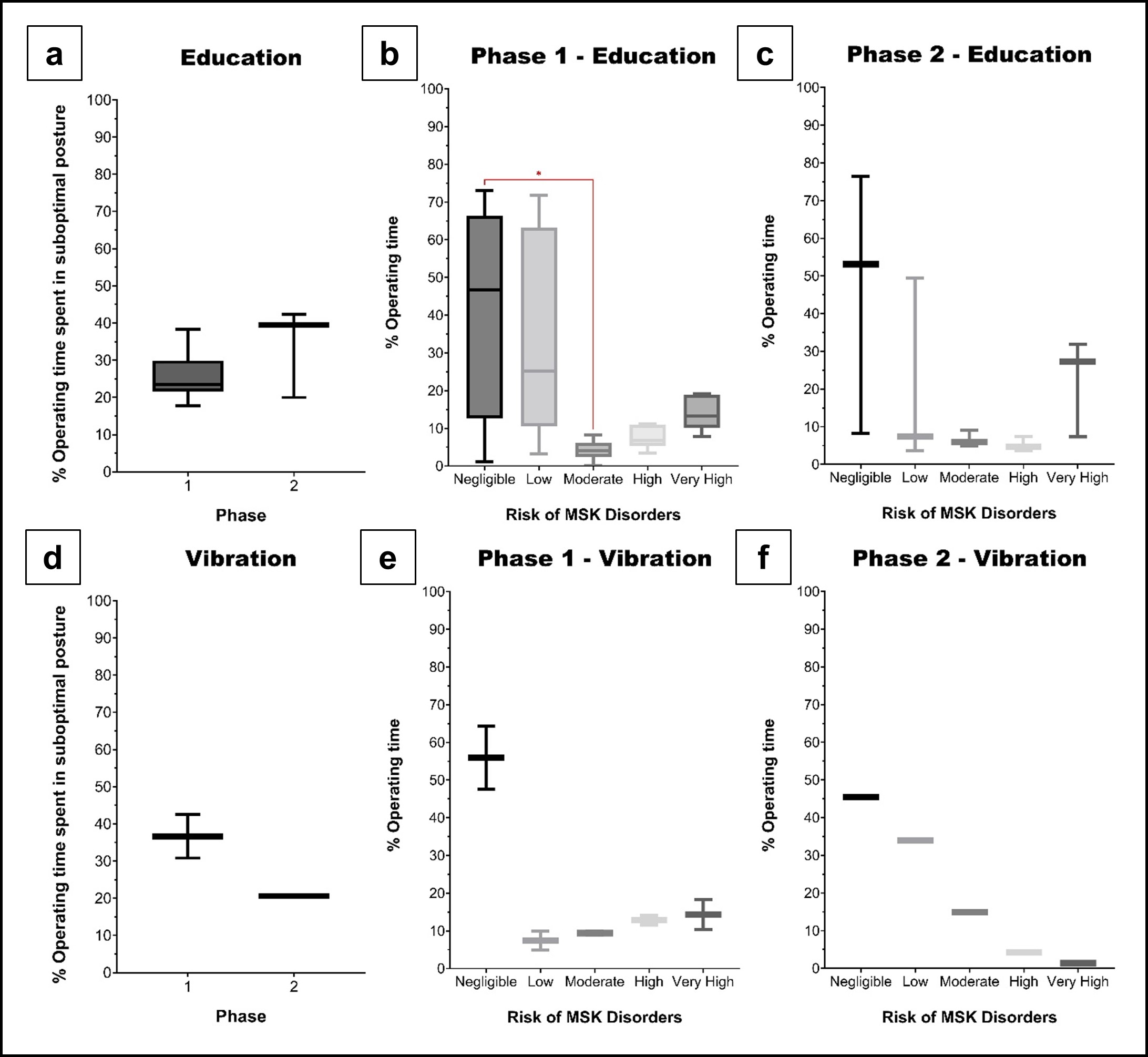

Supplement: Supplementary file 4 — Supplementary file4 (JPG 317 KB) Figure S2: Laparoscopic-only Subset Analysis. Time spent in suboptimal posture in participants receiving an a educational intervention d vibrational intervention. Distribution of musculoskeletal disorder risk amongst participants in the educational b Phase 1, c Phase 2; and vibrational e Phase 1, f Phase 2 intervention for each phase (*p=0.02) [file 464_2024_10933_MOESM4_ESM.jpg]
