# Supplementary material for: Using wearable technology for Posture Regulation to Improve Surgical Ergonomics in the paediatric operating room: the UPRISE trial: a pilot study
Source: Surg Endosc. 2024 Jun 20;38(8):4445–56. doi: 10.1007/s00464-024-10933-5 (PMC11289160; doi:10.1007/s00464-024-10933-5)
Supplement: Supplementary file 3 — Supplementary file3 (DOCX 17 KB) Table S1: Participant Demographics. Discrete data is presented as n (%). p-values were calculated using Mann-Whitney U tests for non-normally distributed data (green). p-values were calculated using unpaired t-tests for normally distributed data (blue) [file 464_2024_10933_MOESM3_ESM.docx]

**Table S1:** Participant Demographics

|  | **ALL**  **(n=8)**  **n (%)** | **EDUCATION**  **(n=4)**  **n (%)** | **VIBRATION**  **(n=4)**  **n (%)** | **Significance**  **(p-value)** |
| --- | --- | --- | --- | --- |
| **Gender**  Male  Female | 3 (37.5%)  5 (62.5%) | 1 (25%)  3 (75%) | 2 (50%)  2 (50%) | ns  ($>$0.99) |
| **Height (cm)**  150 – 159  160 – 169  170 – 179  180 – 189 | 2 (25%)  1 (12.5%)  3 (37.5%)  2 (25%) | 1 (25%)  1 (25%)  1 (25%)  1 (25%) | 1 (25%)  0 (0%)  2 (50%)  1 (25%) | ns  ($>$0.99) |
| **Years of Surgical Experience**  $<$ 5 years  5 – 10 years  11 – 20 years  21 – 30 years  $>$ 30 years | 1 (12.5%)  1 (12.5%)  4 (50%)  2 (25%)  0 (0%) | 0 (0%)  1 (25%)  2 (50%)  1 (25%)  0 (0%) | 1 (25%)  0 (0%)  2 (50%)  1 (25%)  0 (0%) | ns  ($>$0.99) |
| **Common Regions of MSK pain/discomfort**  Neck  Shoulder  Back  Wrist  None | 3 (25%)  4 (50%)  3 (37.5%)  0 (0%)  1 (12.5%) | 2 (50%)  2 (50%)  3 (75%)  0 (0%)  0 (0%) | 1 (25%)  2 (50%)  0 (0%)  0 (0%)  1 (25%) | ns  (0.4) |
| **Frequency of MSK pain/discomfort**  Never  Once daily  ≥ Twice daily  Once weekly  ≥ Twice weekly | 1 (12.5%)  1 (12.5%)  0 (0%)  4 (50%)  2 (25%) | 0 (0%)  1 (25%)  0 (0%)  2 (50%)  1 (25%) | 1 (25%)  0 (0%)  0 (0%)  2 (50%)  1 (25%) | ns  ($>$0.99) |
| **Degree of Interference with Work**  Never  Sometimes  Half the time  Most of the time  Always | 6 (62.5%)  2 (25%)  0 (0%)  0 (0%)  0 (0%) | 3 (75%)  1 (25%)  0 (0%)  0 (0%)  0 (0%) | 3 (75%)  1 (25%)  0 (0%)  0 (0%)  0 (0%) | ns  ($>$0.99) |
| **Frequency of Physical Activity (per week)**  Never  1 – 2 times  3 – 4 times  5 – 7 times | 1 (12.5%)  4 (50%)  2 (25%)  1 (12.5%) | 1 (25%)  2 (50%)  0 (0%)  1 (25%) | 0 (0%)  2 (50%)  2 (50%)  0 (0%) | ns  ($>$0.99) |

*Discrete data is presented as n (%). P-values were calculated using Mann-Whitney U tests for non-normally distributed data (green). P-values were calculated using unpaired t-tests for normally distributed data (blue).*
